# Dispersed Power Production in Terms of the Potential of Briquettes Made from Straw and Willow as Renewable Sources of Energy

**DOI:** 10.3390/ma15155235

**Published:** 2022-07-28

**Authors:** Kamil Roman, Emilia Grzegorzewska, Patrycja Zatoń, Anita Konieczna, Sylwia Oleńska, Kinga Borek, Adam Świętochowski

**Affiliations:** 1 Institute of Wood Sciences and Furniture, Warsaw University of Life Sciences, 166 Nowoursynowska St., 02-787 Warsaw, Poland; k.roman@itep.edu.pl (K.R.); patrycja_zaton@sggw.edu.pl (P.Z.); a.konieczna@itp.edu.pl (S.O.); 2 Institute of Technology and Life Sciences-National Research Institute, 05-090 Pruszków, Poland; sylwia_olenska@sggw.edu.pl (A.K.); k.borek@itp.edu.pl (K.B.); 3 Institute of Mechanical Engineering, Warsaw University of Life Sciences, 166 Nowoursynowska St., 02-787 Warsaw, Poland; adam_swietochowski@sggw.edu.pl

**Keywords:** straw, biomass, energy, briquets

## Abstract

The rapid development of agricultural technologies has triggered new possibilities of using plant waste as fuel. Briquetting plant material is one of the methods of using crop residue as permanent energy carriers. Nevertheless, to maintain the normalised properties of briquettes, their small-scale production should follow an established and well-considered deliberate technological process limiting production costs. The material to be used for energy production should, in particular, be pre-prepared in terms of crushing and moisture content to ensure the right product parameters. The article aims to provide an analysis of briquettes with varied physicochemical parameters to determine and order homogenous groups for selected parameters characteristic for briquettes made from various bioenergy materials. The specific aim of the article required a statistical analysis as a tool for separating the selected factors. An analysis of variance (ANOVA) was involved, together with a post-hoc Duncan test. The analyses demonstrated that the briquette composition, such as bulk value, moisture, and ash content can enhance the briquette quality. In discussion, the straw used was compared with other kinds of agricultural biomass samples and considerable differences were identified. The chemical analysis showed a high content of carbon (from 42.64 to 45.66%) and oxygen (from 47.60 to 49.68%). The percentage share of hydrogen in the chemical structure of the materials accounted for approximately 6%. The ash content found while investigating various straw types ranged from 3.67 to 4.26%, making it lower than reported in the literature. The study also looked at the energetic potential of straw and wood biomass. It was noticed that bioenergetic sources are much potentially higher than the materials used in the traditional power sector. Especially where it concerns an unlimited source that can be provided to the bio-energetic sector. The study is intended to focus the future energy sector on the use of bioenergy in terms of applying straw to energy production purposes.

## 1. Introduction

The ongoing fuel crisis, global sanctions, increasing energy demand, inflation, and disruption to the climate all makes bioenergy production an essential and quite dynamically developing economic trend. Of the many renewable sources of energy, the simplest one, which also requires the least transformation, is definitely biomass as a substance of plant origin [1], derived from the products of waste and agricultural residue [2], forest residue, as well as biodegradable waste [3]. Biomass derived from agricultural and forest residue, as well as biodegradable waste, is a substance falling within a wide range of materials with energy production potential. Most research results found in the literature concern compacting three generally available kinds of biomass: derived from energy crops, wood (sawmill) industry waste, and small material found in the forest. The development of bioenergy production in terms of using the source, facilitates the potential of such sources as an alternative fuel creating local competitiveness and becoming independent from foreign countries. This is particularly true given that, for example, the composition of branches and small pieces of timber left in its original form in forest areas poses a high risk of fire.

An additional operation—briquetting—facilitates an increase in the calorific value and in the bulk value of the product. Briquetting is a form of high-pressure biomass compacting to produce solid biofuel [4,5] that can be used in energy production. Briquette production is related to biofuels based on reusing agricultural and forest waste (wood, sawdust, straw, stems, bark, etc.). One of the key advantages of briquettes is that they are produced naturally and they do not produce any harmful substances to the environment. As indicated by Marreiro et al. in 2021 [6], briquetting ensures solid fuel homogeneity, it facilitates more homogenous burning, it enhances physical and energetic properties, and also facilitates its transport and storage. In addition, biomass briquetting can lower the risk of fires [7]. Another way to produce biomass fuel can be via aerobic fermentation. In the literature [8], the calorific energy values (CEV), the moisture content (MC), and other important parameters can be found. For example, the average CEV of that kind of material can obtain 21.25 ± 0.19 MJ/kg. The extractable oil that contains nitrogen were 17.95 ± 0.02% and 12.80 ± 0.08%, respectively. During research, the XRF analysis confirmed a high amount of carbon and potassium and a low value of mineral content in fermented and dried materials. The literature provides many publications that present the results of studies on biomass acquisition and processing. An important element of these studies is also the ecology aspect, which covers the effect of energy produced from biomass acquired from the environment. In 2011, Aherne et al. [9] investigated the environmental impact of various scenarios of timber acquisition on the status of nutrients in soil and the chemical composition of water in the present and future climate. The authors concluded that only the acquisition of aboveground wood biomass is sustainable and does not exhaust the cation pools in soil long term. They also stressed that, in order to ensure sustainable forest growth, any intensive acquisition of biomass covering the harvesting of whole trees (including the extraction of stumps and roots) will require considering additional nitrogen and potassium inputs.

In 2016, Forsius et al. [10] investigated the possibilities of using energetic wood, while considering the effect of those actions on species diversity, pools of carbon and nutrients in soil, and nutrient leaching. The authors developed scenarios to determine the direction of development of the forest economy in Finland in terms of the use of bioenergy. The studies showed that applying post-harvest residue in heat production decreases the emissions of coal and reduces the absorption of carbon dioxide by forests in the area analysed. Additionally, considering the scenario assuming the maximisation of the use of post-harvest residue for bioenergy production, the biomass of dead stem wood would decrease by approximately 40%.

In 2020, Nurek et al. [11] studied the effect of selected factors on briquette quality. The factors they paid special attention to were briquette moisture content, density, mechanical strength, as well as biomass susceptibility to compaction. The study found a significant dependence between the fraction composition of biomass and its susceptibility to process parameters and the final product quality.

According to the requirements of the European Committee for Standardization (CEN), durability is defined by the durability factor. The standard describes this parameter as a measure of the resistance of biodegradable energy carriers in solid form to shocks or abrasion during transport and handling. The literature contains data on the durability of briquettes made of various types of plant material [12,13]. However, there is no information on the durability of briquettes from mixtures of shredded pine wood residues. The summary of the durability factor (Ψ) of briquettes made of various types of raw materials described in the literature is presented in Table 1.

As reported by Dyjakon et al. in 2020 [14], one of the more important processes of biomass agglomeration is briquetting, and the material undergoing that process shows special performance features. Those special properties of briquettes are mostly related to the right moisture level, an increased bulk value, as well as a higher energy density.

In 2019, Zhang et al. [15] stressed that straw briquette fuel is one of the major possibilities of the use of that material, especially in rural areas. The study showed the organic fertilisers made from straw, as well as replacing the stove coal with straw briquette fuel, can have a considerable effect on energy and on the environment. In addition, straw briquette fuel has been shown to be more acceptable to farmers, which means that it is a solution to the problem of straw burning outdoors.

An extensive literature review of the process of briquetting was presented by Marreiro et al. in 2021 [6]. The authors analysed the results of empirical studies in terms of briquetting process variables and quality parameters of briquette production. They also made a review of the key technologies applied in briquette production, as well as the machinery used. The authors confirmed that the most frequently studied input variables include the biomass particle size, the compacting pressure, the material moisture content, as well as the compacting time. In addition, the analyses of the effect of variables on the briquetting process and briquette properties consider the participation of a binder or temperature. The authors recommend the use of material that shows the highest initial calorific value, which will contribute to a higher final briquette calorific value.

Similarly, Dinesha et al. [16] made a biomass briquetting literature review. The study reported shows that briquette burning characteristics are related with the kind of input, as well as with moisture content, density, a binder content, and with the briquetting method applied. It was confirmed that biomass briquettes can satisfy the energy demand for cooking and heating. This concerns mostly rural areas with a relatively high amount of biomass, which is related with agricultural activity and the occurrence of rural areas. Similarly, numerous studies confirm that a high briquette strength and lifetime requires the right biomass parameters (the kind of the material used, the lignin content, the moisture content, the particle size, and the distribution) [17,18]. More and more frequently, the importance of postproduction factors is also stressed, namely the conditions of storage and transport [19].

## 2. Materials and Methods

### 2.1. Materials

This study analyses three independent briquette lots made from various bioenergy materials. This facilitated a comparative analysis of the samples in terms of their physicochemical properties. The materials selected during the study were commercially available straw briquettes and specially made briquettes made from willow as a native sample. The literature contains information about the influence of individual physical parameters on the calorific energy value. The moisture of the raw material has a significant impact, which should be in the range of 10–15%. Such results were obtained by scientists who, in the course of their research, compacted shredded corn cobs with a moisture content of 10% [20], lupine meal with a moisture content of 13–14% [21], as well as shredded wheat straw, barley straw, corn, and millet with a moisture content of 12–15% [22]. The compaction of grasses to a similar humidity (12 and 15%) was also investigated by another team of scientists [22], determining the density of pellets from crushed wheat straw, barley straw, corn husks, and millet.

The most popular agricultural waste used in Polish renewable energy is straw. In traditional animal husbandry, which was still dominant at the beginning of the 1980s, there was high demand for this raw material, which was used in large amounts as bedding material. According to the literature [23], in Poland, the average annual surplus of straw equalled 12.5 million tonnes (4.2 Mtoe). Although the production of biomass is difficult to estimate, a considerable amount of biomass can be considered as surplus with energy production potential. Additionally, forest residue can also be used as a source of thermal bioenergy due to its high content of carbon and low content of nitrogen and sulphur. The material under study demonstrates lower calorific values, ranging from 15 to 18 MJ/kg [24], as compared with other fuels.

The first product analysed was a straw briquette manufactured by Asket (Poland). The company manufactures its goods based on specially prepared production lines making briquettes made from non-wood biomass for small, medium, and large farms. The producer states that straw briquettes provide a calorific value between 15.5 and 17.5 MJ/kg, where the bulk value reaches up to approximately 650 kg/st. The briquette is 80 mm in diameter and shows ash content from 2 to 4% [24].

The second briquette analysed was a straw product by HolzPower. The product is common on the German market. The briquette was up to 4 cm high and 5 cm in diameter, it was made in a piston press briquetting machine, which is a device quite frequently used for compacting this type of material. The operation of machinery in that system involves feeding the material to the compaction chamber and then pressing the unfinished product with an integrated-with-a-piston hydraulic cylinder. An automatic controller, as part of the compaction system, acquires the set pressure during the operation and allows the briquette to be pushed out of the chamber [25].

The third briquette analysed was a product made from basket willow (*Salix vinimalis*) using a piston press. Following the assumptions, the parameters recorded while briquetting in the compaction head reflected the conditions that occur in professional industrial briquetting presses. The value of the temperature set on the controller corresponded to the temperature of the heater installed and to the values of temperature most often reported in the applicable literature [17,26,27]. The process of briquetting was made for samples with a bulk value of approximately 500 cm^3^. The constant parameter was the internal diameter (Ø45 mm), the length of the compaction chamber (300 mm), and the piston speed (2 mm·s^−1^). These parameters of the compaction head, as well as the maximum compaction force (100 kN), determined the maximum unit pressure, which reached a value of 63 MPa.

If it turns out that straw shows similar parameters to wood biomass, then the approach proposed is to use that material for energy production by applying the process of burning. With that approach in mind, further in the analysis, straw was compared with basket willow to compare with a fuel representing forest biomass. The comparison of both materials will facilitate a comprehensive presentation of energy and economic similarity, determining not only the cost of acquisition and availability, but also the calorific value of the materials selected.

### 2.2. Methods

#### 2.2.1. Statistical Analysis 

During the empirical studies, the material was analysed in terms of the physicochemical properties to determine the factors characteristic for a given lot [28]. The analyses provided the details covering the moisture, ash, and bulk value of respective briquettes. The empirical studies involved a physicochemical analysis of the material to determine the factor properties for further statistical analysis. The statistical analysis facilitated determining the effect of common material factors empirically identified as the values of moisture, ash content, and bulk value depending on the type of production technology. Individual technologies were dedicated to agricultural waste (straw) processing and to energy crops (basket willow *Salix vinimalis*).

After an initial analysis of the materials, the idea was to make a thorough analysis of the straw structure to determine the material quality depending on its availability. With the above in mind, at the second stage of the study the structure of straw microelements was analysed depending on the crop.

The laboratory results required statistical analysis using the ANOVA method. This method helps to determine the effect of variable affiliation of a unit to a specific group on the value of the variable. The variables in the study include chemical parameters and mineral content. The boundary values will be used as factors to provide statistical data after the analyses.

#### 2.2.2. Chemical Analysis of the Tested Biomass 

The content of carbon, hydrogen, nitrogen, and *sulphur* was *analysed using* speciali*s*ed laboratory equipment at Vario macro elementary. Before starting the tests, it was *each time* necessary to perform a calibration by *perform*ing a control test. The device preparation procedure was based on the sterile determination of three samples (blank), during which the device automatically cleans the entire circuit responsible for the sorption of elements. Purging the circuit involves introducing pure oxygen and then helium (60), which flushes out the remaining elements as a pure gas. Rinsing is performed automatically, individually for each measured element (15 rinses of the entire circuit). After the rinses, sulphonamide is introduced to verify the apparatus settings. Sulphonamide is a biologically very stable substance; the constant content of microelements is not subject to the ageing processes. The obtained measurement result is compared with the template. As the accuracy of the measurement is affected by an error of 10–18 mg (a trillion parts), most measurement errors are due to the human factor.

During the actual measurement, the dried raw material was comminuted and micronised. Fully dry and micronised biomass went through a sieve with a mesh diameter of 0.2 m, which allowed for more accurate measurement results to be obtained. The method of testing macroparticles (plant method) assumes the preparation of at least three samples with a maximum mass of 200 mg. Tungsten trioxide catalysts should be added to each of the samples. Tungsten trioxide (WO_3_) is a relatively heavy substance, so the sample volume is small. The total weight of the measured sample in the 1:1 ratio (tested material with catalyst) should not exceed 200 mg. The prepared sample was to be pelleted in a zinc plate with a weight of 73 mg, but the weight of the package was not taken into account during the measurement. The sample was placed in an automatic carousel (automatic sampler). In practice, samples weighing 50 g were prepared, which resulted in a more accurate measurement of the ash value after the oxidation of the raw material. The testing time for one sample was approximately 10 min. After completing the tests, the columns had to be cooled down, as the first one was heated to 1000 °C and the second to 800 °C. 

The analysed research material is not a dedicated energy resource, meaning that the results of the measurements of chemical composition were compared with two other types of biomass that are commonly used in renewable energy. The obtained test results were compared with the literature values for saw shavings and energy chips, assuming that the selected raw materials are chemically the closest to the logging residues among the available biofuels. These raw materials were obtained from heating companies that use biomass in the energy production process. The share of carbon will determine the calorific value of the fuel, while the content of sulphur and nitrogen reflects the pro-ecological features of the material, because combustion causes elements such as sulphur and nitrogen to create undesirable compounds in the form of sulphur oxide (SO_2_) and nitrogen dioxide (NO_2_), the emission of which has a strong effect on the ozone layer.

#### 2.2.3. Moisture Measurement 

The biomass moisture can be measured using several methods. The study measured moisture using the weight difference [29]. The drying time was specific for the sawdust and woodchip samples. The drying process was carried out under laboratory conditions, at a temperature of 105 °C, with an accuracy ±1 °C. The moisture (*W*) of the crushed woodchip residue samples was investigated for the initial material distribution, following the PN-ISO 589:2006 guidelines.

## 3. Results

### 3.1. Preliminary Study 

The objective of the tests was to determine the factors with a significant effect on briquette properties. The analysis was made with a Student’s *t*-test to determine which of the factors had a significant effect on the parameters. The empirical measurements provided data on moisture, ash, and the bulk value of respective briquettes. The parameter breakdown for the respective briquettes is presented in Table 2.

**Figure 1:**
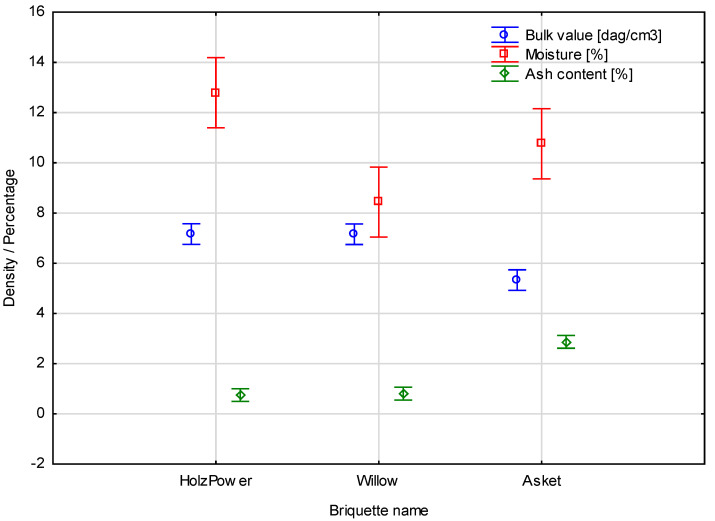
Sums for the briquettes made from various biofuel materials.

During the statistical analysis, the agreed factors were the results of the empirical measurements, though the briquette type and its kind was determined as a quality predictor. The sums for the briquettes made from various biofuel materials are presented in Figure 1.

The Duncan test for varied ash content (%), depending on the specific density, showed a partial dependence creating two homogenous groups. Separator alpha between the individual homogenous groups created was *p* = 0.05. The statistical intergroup error was *MS* = 0.03262. Similarly, the Duncan test for variable moisture (%), depending on specific density, showed a partial dependence creating two homogenous groups. The statistical intergroup error was *MS* = 0.97856. The last measurement was also analysed with the Duncan test. Bulk density (dag/cm^3^), depending on specific density, showed a partial dependence creating two homogenous groups. the statistical intergroup error was *MS* = 0.08334. The characteristics of the separated homogenous samples and the values of the means characteristic for the effect of the parameters measured are presented in Table 3.

After a preliminary analysis of the basic studies, similarities were noted across the parameters characteristic for straw and the parameters characteristic for wood biomass. Considering the limits of the wood biomass, caused by both the global economic situation and the natural limitations to the growth of perennial woody plants the wood biomass is acquired from, the observation provides a springboard for further research using straw.

### 3.2. Main Research 

As part of the main research, the basic elemental composition of straw was determined in order to carry out a qualitative analysis of the material. The elemental composition is one of the basic factors affecting material management in terms of its further use. To compare, the results of the study of the chemical composition are broken down with the data acquired from various kinds of biomass. The material showed differences in chemical compounds, depending on the sampling locations and the composition differences for 24 farms selected. On the farms, the following were cultivated: wheat (four farms), triticale (nine farms), rye (nine farms), and rapeseed (two farms). The material was very diverse, which probably results from the environmental factors of growth and the anisotropic structure of the material. The samples were collected directly from the field during the annual harvesting onto the vehicles, which prevents the biomass from contamination with the mineral fraction.

To determine the calorific value, the heat of combustion, moisture, and the elemental composition of straw, the lab tests were made with the sample of straw of three crop species (wheat, rye, and rapeseed). The samples were properly homogenised and prepared for laboratory analysis to determine the chemical properties, such as total hydrogen, total carbon, total nitrogen, total sulphur, and oxygen (the calculation method). The chemical composition of the biomass is given in Table 4.

**Figure 2:**
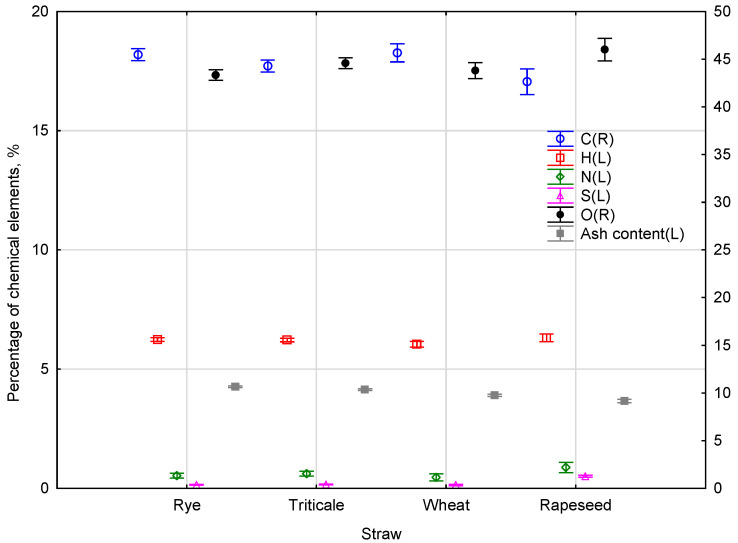
Sum of the chemical composition of straw from various harvesting sources.

The straw from different harvesting sources, in addition to the chemical composition, also contained various types of contamination, with the shares of mineral compounds containing ash, which must have been due to the type of raw material source, given that the straw was taken from different plantations. According to the test results, the carbon content in the material analysed ranged from 41.75 to 46.71%, the hydrogen content from 6.01 to 6.48%, the content of nitrogen from 42.26 to 47.03%, the content of sulphur from 0.14 to 0.55%, the oxygen content from 51.68 to 53.29%, and the ash content from 3.61 to 3.89%. The results were exposed to the statistical analysis to determine the effects of respective parameters on the type of the straw biomass produced. In the figure below, the primary axis (L) shows the value of H, N, S, O, and ash content, with the value of C being placed on the secondary axis (R). The sums for the chemical composition of the straw from various harvesting sources are presented in Figure 2.

The expected marginal means were determined for various straw types, where Wilks’ lambda was 0.00223, while the empirical value of statistics F (15, 44,57) = 24.171. According to the methodology, to confirm the effect of the chemical content of straw on its origin, the Duncan statistical test was made. The characteristics of homogenous groups determining the effect of the parameters measured are given in Table 5.

The ANOVA indicated some differences between the straw types and the Duncan test was used to assign the materials to the homogeneous groups. In every chemical property test, the individual material formed a separate homogeneous group. The most confirmed considerable differences were recorded for the ash content, where individual materials create individual homogeneous groups.

## 4. Discussion

Recently, energy security and cutting greenhouse gases are the key aspects behind the use of agricultural biomass. In Poland, straw is the biomass material of agricultural origin. A straw surplus, given that straw is used as part of agricultural production, can therefore be a source of fuel. Straw is a valuable fuel with a constantly growing demand. Due to a non-homogenous chemical composition and a varied calorific value that depends on moisture, straw is also a fuel that is quite difficult to use for energy purposes. Its non-homogeneity is also affected by growing conditions and the kinds of fertilisation used.

The chemical composition of lignocellulosic biomass is approx. 50% carbon, approx. 43% oxygen, approx. 6% hydrogen, approx. 0.2% nitrogen, and other elements such as iron, sodium, potassium, magnesium, phosphorus, sulphur, and silicon. Chemical compounds that make up both the organic and inorganic structures of matter take the form of simple and complex chains. Carbon, hydrogen, and oxygen most often take the form of hydrocarbons, cellulose, and lignin, and less often in the form of resins, fats, waxes, tannins, and proteins, compounds of which have a significant impact on the course of thickening of plant materials [30].

The chemical analysis showed a high content of carbon (from 42.64 to 45.66%) and oxygen (from 47.60 to 49.68%), which confirms that the material tested is organic. The percentage share of hydrogen in the chemical structure of the materials accounted for approximately 6%. The content roughly corresponded to the limits reported by Friedl et al. [31] for plant materials (approximately 5.9%). The statistical analysis revealed that the differences in the oxygen content across the types of straw were non-significant, though one clearly shows a lower percentage. Other materials belonged to a single homogeneous group. 

The content of carbon, hydrogen, and oxygen in solid biomass depends on the ongoing processes of metamorphism [32]. The ongoing processes do not have such a significant impact on the share of other elements (nitrogen and sulphur). After burning, the elements of a mineral nature remain in the form of ash [33,34].

According to the available literature [31], the nitrogen and sulphur content in that kind of plant material ranges from approximately 0.8% of N and 0.1% of S. The percentages of nitrogen and sulphur in the structure of the material analysed might have been influenced by silvicultural practices, site conditions, soil quality, and by other environmental parameters. As for the type of biomass examined here, the nitrogen and sulphur content ranged from 0.46 to 0.87% for N and 0.14 to 0.51% for S, which was consistent with the data reported in the literature. All the types of biomass considered in this paper revealed low levels of sulphur and nitrogen, besides one. Rapeseed straw has a high sulphur content (approximately 0.51%), as compared with the other types of straw.

In the case of ash content, statistical analysis showed significant differences across various biomass types, by separating the straw types into individual homogeneous groups. According to Friedl et al. [31], the ash content in the plant material should be approximately 4.9%. The ash content found while investigating the different straw types ranged from 3.67 to 4.26%, and it was lower than reported in the literature. The differences in the ash content across the straw types were significant, with the highest value recorded for rye straw (4.26%), which might be due to a high degree of biomass contamination during storage or fertilisation. A positive aspect for the ash produced from straw briquette fuel burning is the possibility of using it as a fertiliser. It should be noted that the materials were collected from various plantation locations, so the differences identified were difficult to account for. 

The mean oxygen content was calculated by subtracting the percentage shares of the other chemical elements and ash. For all the straw types (from 47.60 to 49.68%), the oxygen content was slightly higher than reported in the literature for similar lignocellulosic materials [31,35]. The percentage share of oxygen found here for the residue fell within the range reported for wood derivatives, from 43.20% for spruce wood [36] to 45.6% for birch branches [37]. The Duncan test qualified the parameters under study to two separate homogenous groups. It can be noted that oxygen was the second most abundant element in the samples.

Unprocessed straw is a local source of energy that should be prepared and used as close as possible to the place where it was acquired [38], due to the material transport costs. Theoretically, fossil fuels can be replaced with lignocellulosic biomass, but their energy value will not match the energy value of coal. In Poland, the average annual surplus of straw equalled 12.5 million tonnes (4.2 Mtoe). As the literature reports, the net calorific value of straw materials ranges from 15.5 to 17.5 MJ/kg [24]. Assuming that the energy value of lignocellulosic biomass in its original state is of the value stated in the literature, approximately 206,250 TJ of pure energy can be obtained. The biomass potential favours the European Union’s direction for the use of renewable energy, which is leading to distributed generation and the increased use of by-products and organic waste [23].

Straw briquette burning can take place in a heat boiler, an oven, or in a fireplace. The briquette fuel can be used on a greater scale in heating plants and boiler rooms with dedicated heating systems. Providing the optimal combustion efficiency requires the firebed to be supplied with appropriately prepared material. The preparation usually covers the process of crushing and drying the moist material. Straw burning involves successive stages. In firebeds, the material initially starts to smoulder and glow with no flame effect (contrary to the firebeds with blowing air). At the initial burning stage, straw briquette fuel increases its volume and so some free space must be provided in the combustion chamber. The attainable temperature in the firebed can reach up to 900 °C. Importantly, the flame in the combustion process should be dark yellow in colour, as this allows the temperature to be controlled and limits slagging. During combustion, straw briquette fuel emits an inconsiderable amount of greenhouse gases and CO_2_ [39], unlike traditional coal, where particulate matter can fall within 20–30 metres from the firebed. 

In most cases in Poland, straw is used as an energy material in the local heating systems. The heating season in Poland is quite long, lasting from mid-October to early May. It is therefore necessary to store the material, as straw is a by-product of plant production acquired in the summer months. The storage system has a big effect on the quality of the straw. The exposure of straw to weather conditions can have a considerable deleterious effect, lowering the energy value of the stored material. Additionally, in unfavourable storage conditions, straw can become rotten and self-heated.

## 5. Conclusions

The paper evaluates the performance parameters of various types of straw at various stages of its use for energy production. The study covered the chemical evaluation of the plant material, followed by a statistical measurement of the selected parameters. As part of the discussion, the straw was compared with the data available for other kinds of agricultural biomass and considerable differences were identified. The differences can be due to the non-homogeneity of the biomass or to the place of its acquisition. The comprehensive study shows a powerful commercial potential and may trigger some interest in enterprises operating on the market of preparing biomass for the power sector. The study shed new light on the energy industry in Poland and globally, showing that the potential of biomass made from straw and wood sources is much higher than that of the materials used in the traditional power sector. It also strikes a balance in the energy sector, which can provide considerable amounts of material with the desired physicochemical parameters, in a short time and at a reasonable price.

The study will help focus the attention of the future energy sector on the use of bioenergy in terms of applying straw to energy production purposes. Irrespective of the straw cultivation technology applied, a considerable amount of biomass can be considered a surplus with energy production potential. Next to the macroelements, straw contains approximately 4% of mineral matter. The current information shows that forest residue can be used as a source of thermal energy due to its high content of carbon and low content of nitrogen and sulphur. The material used in the study demonstrates lower calorific values, ranging from 15 to 18 MJ/kg [24], as compared with other fuels. However, considering the availability, the physicochemical parameters, and the way the fuels burn, they can substantially replace culm commonly used in the energy sector.

## Figures and Tables

**Table 1:** The summary of the durability factor (Ψ) of briquettes made of various types of raw materials.

Material	Durability Factor (Ψ)	References
Twisted straw (0°)	0.81	[12]
Twisted straw (5°)	0.93	[12]
Twisted straw (10°)	0.9	[12]
Twisted straw (20°)	0.8	[12]
Fodder corn straw (15 tons, mounted forage harvester)	0.75	[13]
Fodder corn straw (20 tons, mounted forage harvester)	0.81	[13]
Fodder corn straw (15 tons, hammer mill)	0.88	[13]
Fodder corn straw (20 tons, hammer mill)	0.88	[13]

**Table 2:** Breakdown of parameters for respective briquettes.

Briquette Commercial Name	Bulk Value (dag/cm^3^)	Moisture (%)	Ash Content (%)
HolzPower (Straw)	7.19	12.74	0.575
	7.35	12.42	0.657
	6.96	13.22	1.020
Asket (Straw)	5.84	12.25	2.790
	4.94	8.97	2.864
	5.20	11.06	2.961
Willow	7.18	8.33	0.673
	7.12	8.42	1.019
	7.17	8.56	0.728

**Table 3:** Characteristics of the effect of the material type on bulk density for the briquette analysed.

Briquette Name	Bulk Value (dag/cm^3^)	Moisture (%)	Ash Content (%)
	Mean		
HolzPower	7.17 ^a^	12.79 ^c^	0.75 ^f^
Asket	5.33 ^b^	10.76 ^d^	2.87 ^g^
Willow	7.16 ^a^	8.44 ^e^	0.81 ^f^

^a, b, c, d, e, f, g^—homogenous group.

**Table 4:** Chemical composition of the straw from various harvesting sources.

Straw	Content of (%)					Ash Content (%)
	C	H	N	S	O	
Rye	44.14	6.38	0.63	0.16	44.38	4.31
Rye	44.96	6.22	0.57	0.17	43.79	4.29
Rye	45.25	6.29	0.62	0.18	43.38	4.28
Rye	44.52	6.27	0.58	0.15	44.20	4.28
Rye	44.02	6.33	0.52	0.12	44.75	4.26
Rye	46.55	6.21	0.37	0.13	42.48	4.26
Rye	46.71	6.16	0.46	0.17	42.26	4.24
Rye	46.43	6.18	0.57	0.15	42.45	4.22
Rye	46.71	6.12	0.48	0.12	42.36	4.21
Triticale	45.22	6.04	0.51	0.16	43.89	4.18
Triticale	45.32	6.04	0.48	0.17	43.81	4.18
Triticale	43.17	6.18	1.16	0.24	45.10	4.15
Triticale	43.47	6.16	0.56	0.16	45.50	4.15
Triticale	44.63	6.32	0.49	0.17	44.25	4.14
Triticale	44.14	6.32	0.57	0.16	44.68	4.13
Triticale	43.73	6.37	0.57	0.13	45.08	4.12
Triticale	44.76	6.32	0.61	0.12	44.07	4.12
Triticale	44.15	6.22	0.54	0.14	44.83	4.12
Wheat	45.83	6.01	0.53	0.12	43.50	4.01
Wheat	45.44	6.07	0.47	0.16	43.94	3.92
Wheat	45.64	6.01	0.42	0.14	43.90	3.89
Wheat	45.73	6.08	0.42	0.14	43.85	3.78
Rapeseed	41.75	6.14	0.89	0.47	47.03	3.72
Rapeseed	43.52	6.48	0.85	0.55	44.99	3.61

**Table 5:** Chemical composition of the straw from various sources of harvesting.

Straw	Content of (%)					Ash Content (%)
	C	H	N	S	O	
Rapeseed	42.64 ^a^	6.31 ^c^	0.87 ^e^	0.51 ^g^	46,01 ^i^	3.67 ^k^
Triticale	44.29 ^b^	6.22 ^c^	0.61 ^e^	0.16 ^h^	44,58 ^j^	4.14 ^l^
Rye	45.48 ^b^	6.24 ^c^	0.53 ^e^	0.15 ^h^	43,34 ^k^	4.26 ^m^
Wheat	45.66 ^b^	6.04 ^d^	0.46 ^f^	0.14 ^h^	43,80 ^j, k^	3.90 ^n^

^a, b, c, d, e, f, g, h, i, j, k, l, m, n^—homogenous group.

## Data Availability

Not applicable.

## References

[1] Hryniewicz M., Roman K. (2021). Simulations of fuels consumption in the CHP system based on modernised GTD-350 tur-bine engine. J. Water Land Dev..

[2] Hryniewicz M., Strzelczyk M., Helis M., Paszkiewicz-Jasińska A., Steinhoff-Wrzesniewska A., Ro-man K. (2021). Mathematical models use to yield prognosis of perennials on marginal land according to fertilis-ers doses. J. Water Land Dev..

[3] Journal of Law No 261, item 2187 of 19 December 2005 on a Detailed Scope of Obligations of Acquiring and Submitting the Certificates of Origin to Be Cancelled, Making a Compensatory Payment, Purchasing Electrical Energy and Heat Produced from Renewable Sources of Energy, §2 Clause 1. https://www.infor.pl/akt-prawny/DZU.2005.261.0002187,rozporzadzenie-ministra-gospodarki-w-sprawie-szczegolowego-zakresu-obowiazkow-uzyskania-i-przedstawienia-do-umorzenia-swiadectw-pochodzenia-uiszczenia-oplaty-zastepczej-oraz-zakupu-energii-elektryczne.html.

[4] Xu J., Chang S., Yuan Z., Jiang Y., Liu S., Li W., Ma L. (2015). Regionalized techno-economic assessment and policy analysis for biomass molded fuel in China. Energies.

[5] Brunerová A., Roubík H., Brožek M., Herák D., Šleger V., Mazancová J. (2017). Potential of tropical fruit waste biomass for production of bio-briquette fuel: Using Indonesia as an example. Energies.

[6] Marreiro H.M.P., Peruchi R.S., Lopes R.M.B.P., Andersen S.L.F., Eliziário S.A., Rotella Junior P. (2021). Empirical Studies on Biomass Briquette Production: A Literature Review. Energies.

[7] Sahoo K., Bilek E., Bergman R., Mani S. (2019). Techno-economic analysis of producing solid biofuels and biochar from forest residues using portable systems. Appl. Energy.

[8] Mohammad S., Baidurah S., Kamimura N., Matsuda S., Bakar N.A.S.A., Muhamad N.N.I., Ahmad A.H., Dominic D., Kobayashi T. (2021). Fermentation of Palm Oil Mill Effluent in the Presence of *Lysinibacillus* sp. LC 556247 to Produce Alternative Biomass Fuel. Sustainability.

[9] Aherne J., MPosch Forsius M., Lehtonen A., Härkönen K. (2012). Impacts of forest biomass removal on soil nutrient status under climate change: A catchment-based modelling study for Finland. Biogeochemistry.

[10] Forsius M., Akujarvi A., Mattsson T., Holmberg M., Punttila P., Posch M., Liski J., Virkkala R., Vihervaara P. (2016). Modelling impacts of forest bioenergy use on ecosystem sustainability: Lammi LTER region, southern Finland. Ecol. Indic..

[11] Nurek T., Gendek A., Roman K., Dąbrowska M. (2020). The impact of fractional composition on the mechanical properties of agglomerated logging residues. Sustainability.

[12] Fiszer A. (2009). Wpływ wilgotności słomy i temperatury procesu brykietowania na jakość aglomeratu. J. Res. Appl. Agric. Eng..

[13] Niedziółka I., Szymanek M., Zuchniarz A. (2008). Ocena trwałości brykietów wytworzonych z masy roślinnej kukurydzy pastewnej. Inżynieria Rol..

[14] Dyjakon A., Sobol Ł., Krotowski M., Mudryk K., Kawa K. (2020). The Impact of Particles Comminution on Mechanical Durability of Wheat Straw Briquettes. Energies.

[15] Zhang S., Deng M., Shan M., Zhou C., Liu W., Xu X., Yang X. (2019). Energy and environmental impact assessment of straw return and substitution of straw briquettes for heating coal in rural China. Energy Pol..

[16] Dinesha P., Kumar S., Rosen M.A. (2019). Biomass Briquettes as an Alternative Fuel: A Comprehensive Review. Energy Technol..

[17] Kaliyan N., Morey R.V. (2019). Factors affecting strength and durability of densified biomass products. Biomass Bioenergy.

[18] Stelte W., Holm J.K., Sanadi A.R., Barsberg S., Ahrenfeldt J., Henriksen U.B. (2011). A study of bonding and failure mechanisms in fuel pellets from different biomass resources. Biomass Bioenergy.

[19] Brunerová A., Brožek M., Šleger V., Nováková A. (2018). Energy Balance of Briquette Production from Various Waste Biomass. Sci. Agric. Bohem..

[20] Kaliyan N., Morey R.V. (2010). Densification characteristics of corn cobs. Fuel Process. Technol..

[21] Grochowicz J., Andrejko D., Mazur J. (2004). Wpływ wilgotności i stopnia rozdrobnienia na energię zagęszczania i wytrzymałość brykietów łubinowych. MOTROL-Motoryz. I Energetyka Rol..

[22] Mani S., Tabil L.G., Sokhansanj S. (2006). Specific Energy Requirement for Compacting Corn Stover. Bioresour. Technol..

[23] Gradziuk P., Gradziuk B., Trocewicz A., Jendrzejewski B. (2020). Potential of Straw for Energy Purposes in Poland—Forecasts Based on Trend and Causal Models. Energies.

[24] Asket The Manufacturer’s Leaflet. https://zlotywegiel.pl/tag/zloty-wegiel/.

[25] Rembowski Ł. (2007). Brykieciarka Tłokowa (mimośrodowa), Stemplowa, Korbowodowa, Agroenergetyka, s. 1. http://agroenergetyka.pl/?a=article&id=199.

[26] Wang Y., Wu K., Sun Y. (2016). Effects of raw material particle size on the briquetting process of rice straw. J. Energy Inst..

[27] Chen W.H., Kuo P.C. (2010). A study on torrefaction of various biomass materials and its impact on lignocellulosic structure simulated by a thermogravimetry. Energy.

[28] Roman K., Roman M., Szadkowska D., Szadkowski J., Grzegorzewska E. (2021). Evaluation of Physical and Chemical Pa-rameters According to Energetic Willow (*Salix viminalis* L.) Cultivation. Energies.

[29] Roman K., Barwicki J., Hryniewicz M., Szadkowska D., Szadkowski J. (2021). Production of Electricity and Heat from Bio-mass Wastes Using a Converted Aircraft Turbine AI-20. Processes.

[30] Thomas M., Vliet T., Poel AF B. (1998). Physical quality of pelleted animal feed 3. Contribution of feedstuff components. Anim. Feed. Sci. Technol..

[31] Friedl A., Padouvas E., Rotter H., Varmuza K. (2005). Prediction of heating values of biomass fuel from elemental composition. Anal. Chim. Acta.

[32] Kraszkiewicz A. (2009). Analiza wybranych właściwości chemicznych drewna i kory robinii akacjowej (*Robinia pseudoacacia* L.). Inżynieria Rol..

[33] Prosiński S. (1984). Chemia Drewna.

[34] Rybak W. (2006). Spalanie i Współspalanie Biopaliw Stałych. Wyd.

[35] Roman K. (2017). Dobór Parametrów Technicznych Procesu Brykietowania Biomasy Leśnej. Ph.D. Thesis.

[36] Kajda-Szcześniak M. (2013). Evaluation of the basic properties of the wood waste and woodbased wastes. Arch. Waste Manag. Environ. Prot..

[37] Bach Q., Chen W., Chu Y., Skreiberg Ø. (2016). Predictions of biochar yield and elemental composition during torre-faction of forest residues. Bioresour. Technol..

[38] Raslavičius L., Grzybek A., Dubrovin V. (2011). Bioenergy in Ukraine—Possibilities of rural development and opportunities for local communities. Energy Policy.

[39] Konieczna A., Roman K., Borek K., Grzegorzewska E. (2021). GHG and NH3 Emissions vs. Energy Efficiency of Maize Production Technology: Evidence from Polish Farms; a Further Study. Energies.

